# Regional variations in serotype distribution and vaccination status in children under six years of age with invasive pneumococcal disease in Germany

**DOI:** 10.1371/journal.pone.0210278

**Published:** 2019-01-09

**Authors:** Stephanie Perniciaro, Matthias Imöhl, Christina Fitzner, Mark van der Linden

**Affiliations:** 1 Department of Medical Microbiology, German National Reference Center for Streptococci, University Hospital (RWTH) Aachen, Germany; 2 Department of Medical Statistics, University Hospital (RWTH) Aachen, Germany; University of Malaya, MALAYSIA

## Abstract

**Overview:**

The protective effect of infant pneumococcal conjugate vaccine (PCV) recommendation can be seen in Germany as a whole and in smaller regional groups. Comparisons between population-normalized geographic regions of Germany show different serotype distributions after program implementation, particularly in non-vaccine serotypes. The prior distinct differences in serotype distribution in children between the former East and former West German federal states have vanished. Children under six remain a vulnerable group, but the occurrence of vaccine-type (VT) invasive pneumococcal disease (IPD) in children correctly vaccinated (using a three-dose primary series plus one booster dose) with PCV13 was low (9 out of 374 cases, 2.4%). However, only 18.4% of children in Germany with IPD were correctly vaccinated with PCV13 according to the recommended schedule. Continued surveillance and better schedule adherence are essential to definitively establish the most effective PCV administration schedule.

**Vaccination effects:**

For all PCV products used in Germany (PCV7, PCV10, and PCV13), vaccination status was the most common statistically significant predictor of infection with a particular serotype: Unvaccinated children old enough to have received at least one dose of vaccine in the PCV7 group had significantly higher odds (OR: 6.84, 95%CI: 2.66–22.06, adjusted for per capita income and residence in the northeastern federal states) of contracting VT IPD. In the PCV10 group, VT IPD had an OR of 4.52 (95% CI: 1.60–15.62, adjusted for year of infection, median household size, and residence in the southern federal states) in unvaccinated children, and in the PCV13 group, unvaccinated children continued to have higher odds (OR: 6.21, 95%CI: 3.45–11.36, adjusted for year of infection, age of child, per capita income, residence in the southern federal states, and percentage of children using public daycare) of getting vaccine-type IPD. Being unvaccinated was the most frequent significant indicator for infection with vaccine-type serotypes for each analysis group, while geographic groupings showed more limited potential to predict serotype of infection in early childhood IPD in Germany.

## Introduction

Invasive pneumococcal disease (IPD) is responsible for nearly half a million deaths per year in children under five, and also represents 5% of all-cause child mortality [1]. The German National Reference Center for Streptococci (GNRCS) has been collecting invasive pneumococcal isolates from children since 1997. Disease surveillance on pediatric IPD is ongoing throughout the world, with a notable uptick following the development of pneumococcal conjugate vaccines (PCVs), which are a common component of childhood immunization programs [2].

Three PCV products have been licensed in Germany: PCV7, PCV10, and PCV13, the last of which replaced PCV7. PCV13 currently has the vast market share of infant pneumococcal vaccination in Germany [3]. The choice of vaccine products, as well as the decision to vaccinate, is made by the parents (in consultation with the pediatrician). The Standing Committee on Vaccinations of the Robert Koch Institute (STIKO) issued a recommendation for all infants to receive the pneumococcal conjugate vaccine in 2006 (a recommendation for premature, chronically ill, or immunodeficient children was made in 2001 [4]), using a 3+1 (third, and fourth, and fifth month of life, with a booster at 11–14 months) schedule [5], and in August 2015, announced a change to a 2+1 dosing schedule (third and fifth months of life, with a booster at 11–14 months) [6]. Here we describe the impact of vaccination status, as well as geographic and demographic factors, on IPD cases for each of the three PCVs over three age cohorts, and examine the changes in serotype distribution in four population-normalized geographic analysis groups.

## Methods

All isolates sent to the GNRCS were identified and serotyped with Neufeld’s Quellung reaction as described elsewhere [7]. Information on vaccination status was either included on the GNRCS questionnaire that accompanies the isolate, or retrieved by telephone follow-up by the GNRCS staff. No identifying information was stored at any time by the GNRCS.

**Fig 1:**
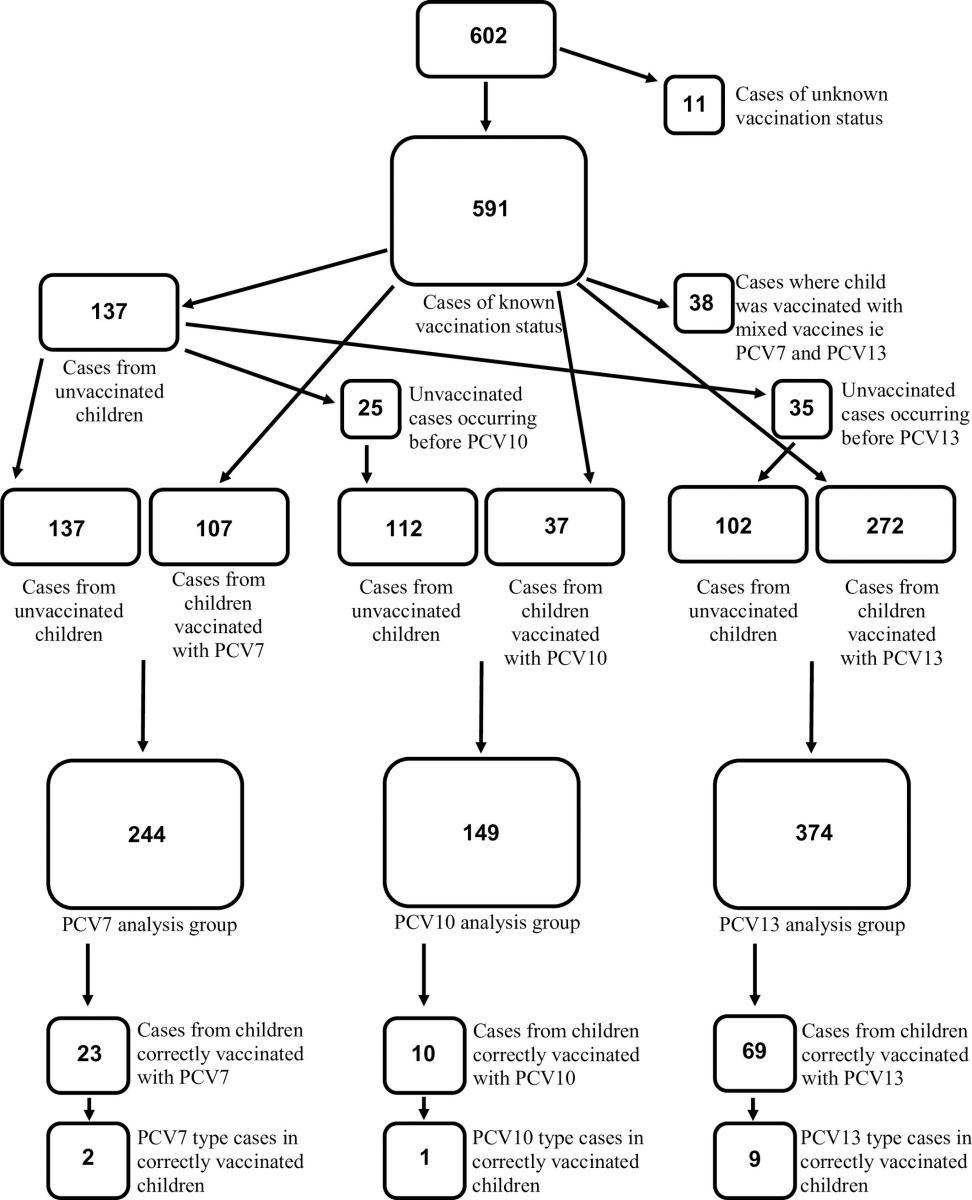
Flowchart of IPD cases included in regression analysis. At the top, all cases of IPD in Germany occurring in children between 90 days and 6 years of age, born after the start of the study period, July 1, 2007 to June 30, 2015, fit the study criteria (n = 602). Depending on vaccination status, year of infection, and age of child, cases were grouped for analysis into a PCV7 group (n = 244), a PCV10 group (n = 149), and a PCV13 group (n = 374). Also shown are all IPD cases from children in each of the respective groups, and vaccine-type IPD cases from children in each group.

**Fig 2:**
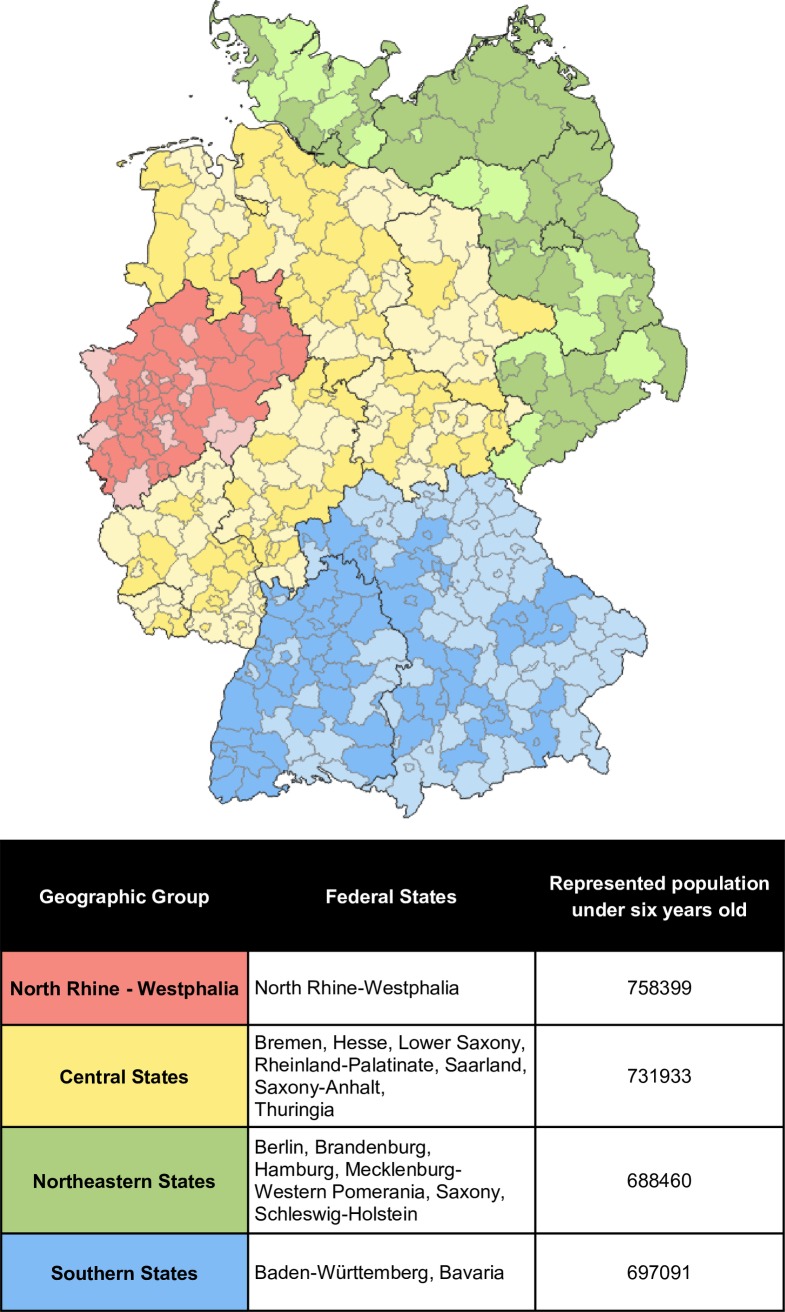
Population-based geographic analysis groups of children under 6 in Germany. Population under 6 years of age based on data from the German Federal Statistical Office for the 31^st^ of December, 2015. Darker shades show districts which submitted at least one IPD isolate to the GNRCS during the study period, July 1, 2007 to June 30, 2015.

IPD cases from children younger than six years of age (minimum age of 90 days) residing in Germany who were born after vaccination program onset (July 1, 2007) were included in the analysis to ensure that all children in the post-vaccination period were within the age window to have been vaccinated in infancy. A flowchart describing IPD cases included in the study analysis and the study population breakdown can be found in **Fig 1**. The 16 federal states of Germany were combined into four geographic analysis groups based on the population of children under six residing in districts which had ever sent a sample to the GNRCS during the study period, shown in **Fig 2**. Each geographic group corresponds to approximately 700,000 children.

The pneumococcal seasons July 2007 through June 2015 are covered by the same vaccine schedule recommendation (three primary doses plus one booster dose). Later seasons were excluded due to the change in vaccine schedule recommendation (to a 2+1 schedule). Vaccination age cohorts are defined as follows: at least one dose (≥90 days old), post-primary series (between 150 and 449 days old), and post-booster (>449 days old).

Vaccination status was separated as follows: “unknown” (excluded from analysis), “unvaccinated” (child had not received any doses of vaccine at time of infection), “vaccinated” (child had received at least one dose of vaccine at time of infection), and “correctly-vaccinated” (child had received the age-appropriate number of vaccine doses within 14 days of the prescribed timeframe). Children who received mixed doses (ie one dose of PCV7 and one dose of PCV13) were excluded from analysis. That is to say, children in the respective analysis groups (PCV7, PCV10, and PCV13) were either vaccinated exclusively with the specified vaccine or wholly unvaccinated.

Multivariate models using Firth’s bias-reduced logistic regression [8] were designed with invasive infection from a specified serotype or specified serotypes fixed as the outcome variable and vaccination status, age of child, year (divided in pneumococcal seasons from July 1^st^ to June 30^th^) of infection, geographic group of residence, residence in the former East German federal states (Brandenburg, Mecklenburg-Western Pomerania, Saxony, Saxony-Anhalt, Thuringia) versus the former West German federal states (Baden-Württemberg, Bavaria, Bremen, Hesse, Hamburg, Lower Saxony, North Rhine-Westphalia, Rhineland-Palatinate, Schleswig-Holstein, Saarland) as predictor variables. Regional district-level demographic data from the 2011 census for median household size, per capita income, percentage of adults without secondary education, percentage of unemployment, and percentage of children under six years of age enrolled in public daycare were added as additional predictor variables. Models were also run with the vaccination status as the outcome variable and the demographic and geographic variables as the predictor variables.

Univariate models were constructed first, for all vaccine type (VT) serotypes in groups, and for single serotypes with at least 10 occurrences [9] in the study period. Predictor variables with P ≤0.20 were selected for the multivariate models. Multivariate models were constructed using forward stepwise logistic regression and McFadden’s pseudo R^2^ to estimate model fit. Multiple logistic regression models were run separately for each age cohort and each vaccine type. For multiple regression models, 95% confidence intervals (95%CIs) of the odds ratio (OR) which did not cross one were considered statistically significant.

Differences in the proportion of serotype distribution and differences in the proportion of vaccinated versus unvaccinated cases across the geographic groups were measured with Fisher’s exact test, with a Dunn- Šidák correction for multiple testing, yielding a significance threshold of 0.002.

All analyses were performed with R (version 3.4.0, The R Foundation for Statistical Computing, 2015). Map graphics were created with QGIS (Quantum Geographic Information System 2017. Open Source Geospatial Foundation Project) using shapefiles from the Global Administrative Areas database; bar graphs and tables were made with Microsoft Excel 2016 and Microsoft Publisher 2016.

## Results

**Fig 3:**
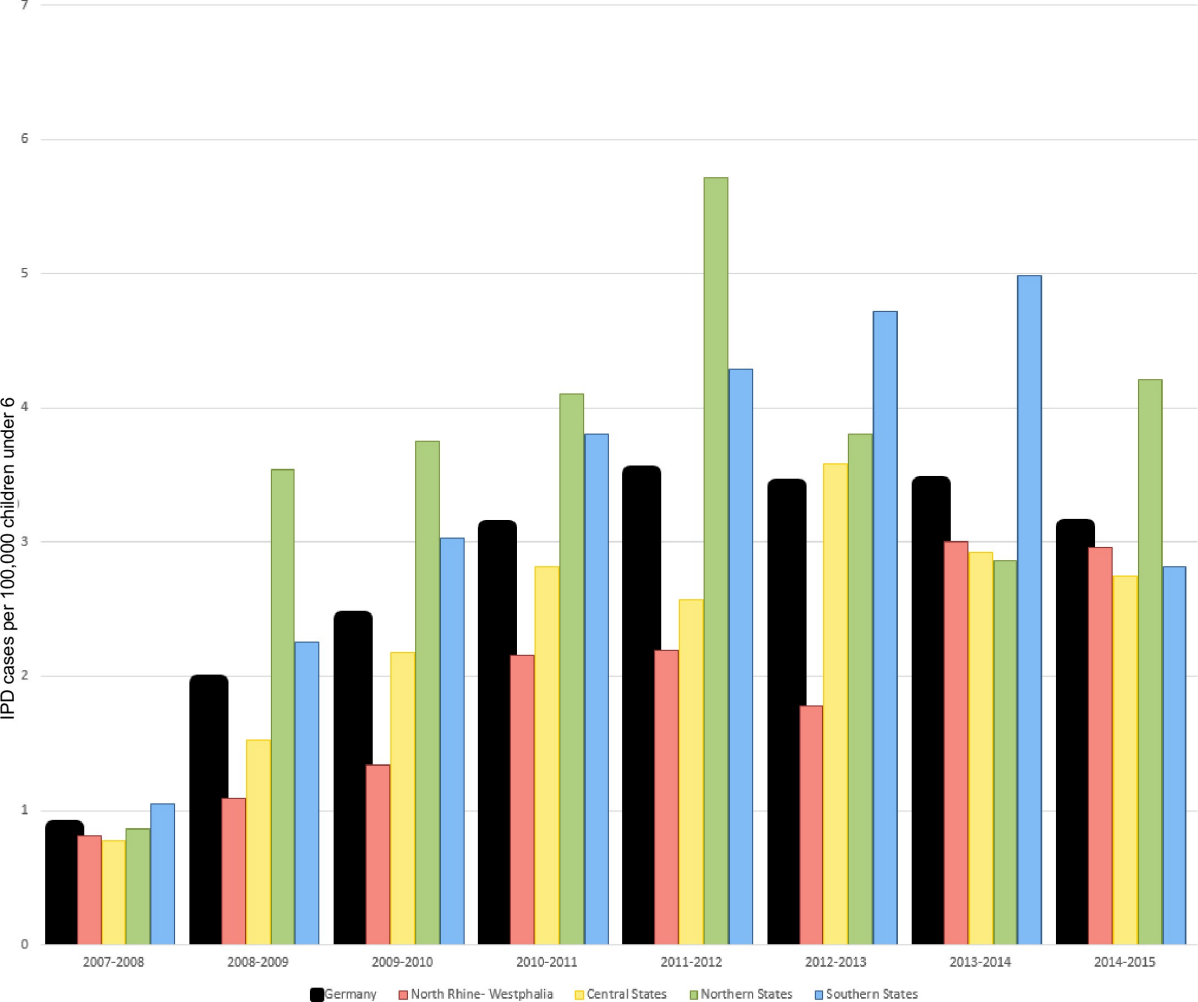
IPD cases in children younger than 6 received by the GNRCS over the study period, 2007–2015. Cases of IPD per 100,000 residents under 6 years of age are shown. Only cases with a known pneumococcal vaccination status are included.

The sample collection of the GNRCS is generated by voluntarily-participating hospital and commercial microbiological laboratories throughout Germany. For the study population, the GNRCS received samples from 162 different laboratories over the study period. The distribution of samples with a known vaccination status received from each geographic group per 100,000 residents under six years of age during the study seasons is shown in **Fig 3**. When separated by geographic group, case numbers ranged between 3 (the 2007–2008 season in the northeastern states) and 33 (the 2014–2015 season in the southern states) during the post-vaccination period. The median age of infection was 404 days (13 months).

**Fig 4:**
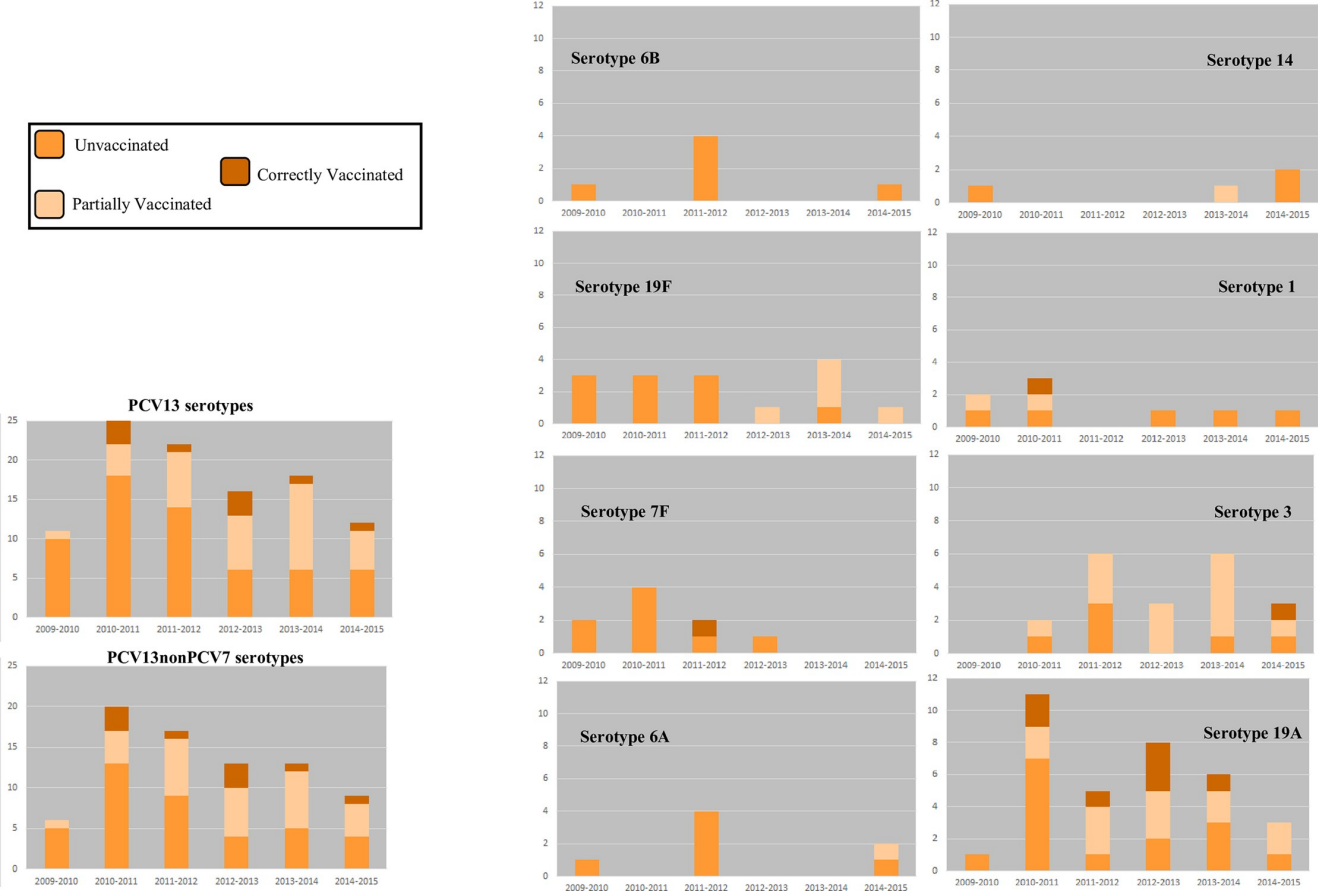
Persistence of Vaccine Serotypes in IPD following implementation of PCV13. Vaccine-type cases of IPD in children under six with a known vaccination status, who received either only PCV13 or no vaccine at all are shown. No IPD cases from serotypes 5, 23F, or 4 occurred during the PCV13 period (2009–2010 through 2014–2015). One case of serotype 18C IPD occurred in the 2010–2011 season, and one case of serotype 9V IPD occurred in the 2011–2012 season, both of which were in unvaccinated children.

A clear vaccination status was established for 71% (591/832) of IPD cases in children over 90 days and less than six years old born after the onset of vaccination. Of these children, 137 (23.2%) were unvaccinated and 454 (76.8%) had received at least one dose of any PCV. Among cases for which the vaccination status was known, unvaccinated children ranged from 19.5% in North Rhine-Westphalia to 29.2% in the southern states; vaccinated children ranged from 70.8% in the southern states to 80.5% in North Rhine-Westphalia. Persistence of vaccine type serotypes is shown in **Fig 4**. Vaccination status in Germany as a whole, by geographic analysis group, and by former political division can be seen in **Table 1**. Multivariate models describing variables associated with vaccination status can be found in **Table 2**. For the PCV7 group, being unvaccinated was associated with the year of infection, and being correctly vaccinated was positively associated with the percentage of adults without a secondary education. No demographic variables reached statistical significance in the PCV10 group, and in the PCV13 group, increasing per capita income was associated with being unvaccinated, and in the post-booster cohort, residence in the southern federal states was associated with being unvaccinated.

**Table 1:** Vaccination status of children with IPD in Germany, 2007–2015.

	Overall				PCV7 group				PCV10 group				PCV13 group			
	n	Not Vaccinated	Vaccinated At All	Correctly Vaccinated	n	Not Vaccinated	Vaccinated At All	Correctly Vaccinated	n	Not Vaccinated	Vaccinated At All	Correctly Vaccinated	n	Not Vaccinated	Vaccinated At All	Correctly Vaccinated
**Germany**	591	137	454	107	244	137	107	23	149	112	37	7	374	102	272	69
		(23.2%)	(76.8%)	(18.1%)		(56.1%)	(43.9%)	(9.4%)		(75.2%)	(24.8%)	(4.7%)		(27.3%)	(72.7%)	(18.4%)
**North Rhine-Westphalia**	113	22	91	19	42	22	20	3	20	16	4	0	73	15	58	13
		(19.5%)	(80.5%)	(16.8%)		(52.4%)	(47.6%)	(7.1%)		(80.0%)	(20.0%)	(0.0%)		(20.5%)	(79.5%)	(17.8%)
**Central States**	150	31	119	30	58	31	27	7	40	27	13	3	93	25	68	19
		(20.7%)	(79.3%)	(20.0%)		(53.4%)	(46.6%)	(12.1%)		(67.5%)	(32.5%)	(7.5%)		(26.9%)	(73.1%)	(20.4%)
**Northeastern States**	150	32	118	34	59	32	27	8	35	25	10	3	95	24	71	21
		(21.3%)	(78.7%)	(22.7%)		(54.2%)	(45.8%)	(8.5%)		(71.4%)	(28.6%)	(8.6%)		(25.3%)	(74.7%)	(22.1%)
**Southern States**	178	52	126	24	85	52	33	5	54	44	10	1	113	38	75	16
		(29.2%)	(70.8%)	(13.5%)		(61.2%)	(38.8%)	(5.9%)		(81.5%)	(18.5%)	(1.9%)		(33.6%)	(66.4%)	(14.2%)
**Former East Germany**	109	21	88	26	41	21	20	6	24	17	7	2	68	16	52	15
		(19.3%)	(80.7%)	(23.9%)		(51.2%)	(48.8%)	(14.6%)		(70.8%)	(29.2%)	(8.3%)		(23.5%)	(76.5%)	(22.1%)
**Former West Germany**	458	109	349	79	190	109	81	17	117	88	29	5	290	79	211	52
		(23.8%)	(76.2%)	(17.2%)		(57.4%)	(42.6%)	(8.9%)		(75.2%)	(24.8%)	(4.3%)		(27.2%)	(72.8%)	(17.9%)

Vaccination status and percentage for each PCV are shown for Germany as a whole, for the geographic analysis groups, and for Former East and Former West Germany.

**Table 2:** Variables influencing pneumococcal vaccination status.

Vaccine	Age Cohort	Vaccination Status	Significant Predictor Variable	Other variables included in the multivariate model
**PCV7**	at least one dose	**NO**	Year of Infection	*Age of Child*
	at least one dose	**CORRECT**	No Secondary Education	*Year of Infection*, *Residence in Former East Germany*
**PCV13**	at least one dose	**NO**	Income per capita	*Residence in North Rhine-Westphalia*, *Residence in the Northeastern Federal States*
	post-primary series	**CORRECT**	Daycare Use	*Residence in North Rhine-Westphalia*, *Residence in the Southern Federal States*, *Income per capita*
	post-booster dose	**NO**	Residence in the Southern Federal States	*Residence in the Central Federal States*

Multivariate logistic regression results describing associations of demographic and geographic variables with pneumococcal vaccination status in children under 6 with IPD in Germany. No variables were significantly associated in the PCV10 group. Complete results with ORs and 95% for univariate and multivariate models can be seen in **S1 Table**.

Multivariate logistic regression results for models exploring factors that influence IPD caused by particular serotypes which had at least one variable reaching statistical significance can be found in **Tables 3, 4** and **5**.

**Table 3:** Multivariate logistic regression results for the PCV7 group.

Age Cohort	Serotype(s) of Infection	Significant Predictor Variable(s)	Other variables included in the multivariate model
at least one dose	**PCV7 types**	Unvaccinated, Residence in the Northeastern Federal States*	*Income per capita*
	**15B**	Residence in North Rhine-Westphalia	*Unvaccinated*
	**15C**	Residence in the Southern Federal States*	*Age of Child*, *Unemployment*, *Residence in North Rhine-Westphalia*
	**19A**	Residence in Former East Germany, Median Household Size*	*Residence in the Northeastern Federal States*
	**19F**	Unvaccinated	*Residence in the Northeastern Federal States*, *Residence in the Central Federal States*
	**23B**	Residence in Former East Germany	*Unvaccinated*, *Year of Infection*
	**38**	Daycare Use*	*No Secondary Education*
	**6B**	Residence in the Southern Federal States	*Unvaccinated*, *Unemployment*, *Income per capita*, *Age of Child*
post-primary series	**10A**	Income per capita	*No Secondary Education*
	**12F**	Correctly Vaccinated	*Year of Infection*, *Age of Child*
	**15C**	Income per capita*	*Residence in the Central Federal States*
	**19A**	Median Household Size*	*Age of Child*, *Residence in Former East Germany*
	**19F**	Unvaccinated, Daycare Use	*Residence in the Central Federal States*
	**24F**	Age of Child	*Residence in North Rhine-Westphalia*
post-booster dose	**PCV7 types**	Unvaccinated	*Residence in the Southern Federal States*, *Daycare Use*
	**1**	Residence in the Central Federal States, Age of Child	*Unvaccinated*, *North Rhine-Westphalia*, *Daycare Use*
	**19A**	Median Household Size*	*Correctly Vaccinated*, *Residence in Former East Germany*, *Residence in the Northeastern Federal States*, *Year of Infection*, *Age of Child*
	**24F**	Year of Infection	*No Secondary Education*
	**38**	Daycare Use*	*Residence in the Central Federal States*, *Age of Child*

Results are divided into three age cohorts: old enough to receive one dose of vaccine (≥90 days), old enough to receive the full primary series (150–449 days old), and old enough to receive the booster dose (>449 days old). * = significant negative association between the listed variables (OR<1). Full results including ORs and 95% CIs for univariate and multivariate models are shown in **S2 Table**.

**Table 4:** Multivariate logistic regression results for the PCV10 group.

Age Cohort	Serotype(s) of Infection	Significant Predictor Variable(s)	Other variables included in the multivariate model
**at least one dose**	**PCV7 types**	Median Household Size	*Residence in the Northeastern Federal States*, *Income per capita*
	**PCV10 types**	Unvaccinated, Year of Infection*, Median Household Size	*Residence in the Southern Federal States*
	**PCV10non7 types**	Unvaccinated	*Year of Infection*
	**10A**	Correctly Vaccinated	*Age of Child*, *Residence in the Northeastern Federal States*, *Residence in the Southern Federal States*, *Unemployment*
	**12F**	Year of Infection	*Unvaccinated*, *Residence in the Central Federal States*, *Residence in the Northeastern Federal States*, *Unemployment*
	**15B**	Correctly Vaccinated	*Residence in the Central Federal States*
	**19F**	Year of Infection*, Daycare Use	* *
	**7F**	Year of Infection*	*Unvaccinated*, *Residence in the Central Federal States*, *Unemployment*
**post-primary series**	**PCV10 types**	Unvaccinated	*Income per capita*, *Year of Infection*, *Residence in Former East Germany*, *Daycare Use*
	**14**	Year of Infection	*Residence in North Rhine-Westphalia*
	**15B**	Correctly Vaccinated	*Residence in the Central Federal States*, *Residence in the Northeastern Federal States*
	**19F**	Year of Infection*, Daycare Use	
	**6A**	Median Household Size*	*Income per capita*
**post-booster dose**	**19A**	Residence in North Rhine-Westphalia, Median Household Size*	*Year of Infection*, *Age of Child*
	**24F**	Year of Infection	*Residence in North Rhine-Westphalia*
	**3**	Correctly Vaccinated	*Age of Child*, *Daycare Use*

Results are divided into three age cohorts: old enough to receive one dose of vaccine (≥90 days). old enough to receive the full primary series (150–449 days old). and old enough to receive the booster dose (>449 days old). * = significant negative association between the listed variables (OR<1). Full results including ORs and 95% CIs for univariate and multivariate models are shown in **S3 Table**.

**Table 5:** Multivariate logistic regression results for the PCV13 group.

Age Cohort	Serotype(s) of Infection	Significant Predictor Variable(s)	Other variables included in the multivariate model
**at least one dose**	**PCV7 types**	Unvaccinated	*Year of Infection*, *Residence in North Rhine-Westphalia*, *Residence in the Northeastern Federal States*, *Residence in the Southern Federal States*
	**PCV10 types**	Unvaccinated, Year of Infection*	*Residence in the Southern Federal States*
	**PCV13 types**	Unvaccinated, Year of Infection*, Age of Child	*Residence in the Southern Federal States*, *Income per capita*, *Daycare Use*
	**PCV13non7 types**	Unvaccinated, Year of Infection*, Age of Child	* *
	**PCV13non10 types**	Unvaccinated, Age of Child	*Year of Infection*, *Residence in the Central Federal States*, *Residence in the Southern Federal States*, *Median Household Size*
	**10A**	Correctly Vaccinated	*Year of Infection*, *Age of Child*, *Residence in the Southern Federal States*, *Unemployment*, *Median Household Size*
	**12F**	Year of Infection, Residence in the Northeastern Federal States, Unemployment, Daycare Use	*Residence in North Rhine-Westphalia*
	**15C**	Age of Child	*Correctly Vaccinated*, *Residence in the Central Federal States*, *Unemployment*
	**19F**	Unvaccinated	*Year of Infection*, *Age of Child*, *Residence in the Central Federal States*, *Residence in the Northeastern Federal States*, *Residence in the Southern Federal States*
	**24F**	Unvaccinated*	*Year of Infection*, *Residence in North Rhine-Westphalia*, *Unemployment*
	**3**	Age of Child	*Correctly Vaccinated*, *Residence in the Southern Federal States*
	**38**	Residence in North Rhine-Westphalia	*Unvaccinated*
	**6A**	Unvaccinated, Age of Child	*Residence in the Central Federal States*, *Residence in the Southern Federal States*, *Income per capita*
	**6B**	Unvaccinated	*Year of Infection*, *Age of Child*, *Residence in the Southern Federal States*, *Unemployment*, *Income per capita*
	**7F**	Unvaccinated, Year of Infection*	*Age of Child*, *Residence in the Central Federal States*, *Unemployment*
**post-primary series**	**PCV7 types**	Unvaccinated	*Year of Infection*, *Age of Child*
	**PCV10 types**	Unvaccinated	*Year of Infection*, *Age of Child*, *Residence in North Rhine-Westphalia*, *Residence in Former East Germany*
	**PCV13 types**	Unvaccinated, Year of Infection*	*Residence in North Rhine-Westphalia*, *Residence in Former East Germany*
	**PCV13non7 types**	Unvaccinated	*Year of Infection*, *Residence in the Northeastern Federal States*
	**PCV13non10 types**	Unvaccinated	*Year of Infection*, *Age of Child*
	**10A**	Correctly Vaccinated, Year of Infection	*Median Household Size*
	**12F**	Daycare Use	*Age of Child*, *Residence in North Rhine-Westphalia*, *Residence in the Northeastern Federal States*
	**14**	Unvaccinated, Year of Infection	
	**19A**	Unvaccinated, Age of Child	*Year of Infection*
	**19F**	Year of Infection*	*Unvaccinated*, *Residence in North Rhine-Westphalia*, *Residence in the Southern Federal States*
	**24F**	Unvaccinated*, Income per capita	
	**6A**	Median Household Size*	*Unvaccinated*, *Year of Infection*, *Residence in Former East Germany*
**post-booster dose**	**PCV7 types**	Age of Child	*Unvaccinated*, *Residence in the Southern Federal States*, *Residence in Former East Germany*, *Daycare Use*
	**PCV10 types**	Age of Child	*Unvaccinated*, *Year of Infection*, *Residence in North Rhine-Westphalia*, *Residence in Southern Federal States*, *Residence in Former East Germany*, *Daycare Use*
	**PCV13 types**	Year of Infection*, Age of Child	*Unvaccinated*, *Residence in the Central Federal States*, *Residence in the Southern Federal States*, *Residence in Former East Germany*, *Income per capita*, *Daycare Use*
	**PCV13non7 types**	Year of Infection*, Age of Child	*Unvaccinated*, *Residence in the Central Federal States*, *Residence in the Southern Federal States*, *Income per capita*, *Daycare Use*
	**PCV13non10 types**	Residence in the Central Federal States*	*Unvaccinated*, *Year of Infection*, *Residence in the Southern Federal States*, *Residence in Former East Germany*, *Median Household Size*, *Daycare Use*
	**15C**	Correctly Vaccinated	*Year of Infection*
	**19A**	Median Household Size*	*Year of Infection*, *Income per capita*
	**19F**	Age of Child	*Daycare Use*
	**24F**	Year of Infection	*Residence in North Rhine-Westphalia*
	**6A**	Residence in the Southern Federal States	*Unvaccinated*, *Age of Child*, *Income per capita*

Results are divided into three age cohorts: old enough to receive one dose of vaccine (≥90 days). old enough to receive the full primary series (150–449 days old). and old enough to receive the booster dose (>449 days old). * = significant negative association between the listed variables (OR<1). Full results including ORs and 95% CIs for univariate and multivariate models are shown in **S4 Table**.

In the PCV7 group (n = 244), the OR for invasive infection with a VT serotype was 6.84 in unvaccinated children for children old enough to receive at least one dose, while the OR for serotype 19F was 7.17. In the post-primary series cohort (n = 110), unvaccinated children had significantly higher odds of contracting serotype 19F IPD, while correctly-vaccinated children had higher odds of contracting Serotype 12F IPD. In the post-booster cohort (n = 88), odds of VT IPD were significantly higher in unvaccinated children. In the PCV7 group, in addition to vaccination status, residence in the northeastern federal states, residence in North Rhine- Westphalia, residence in the southern federal states, residence in former East Germany, median household size, and percentage of children enrolled in public daycare were statistically significant predictor variables for at least one serotype.

The PCV10 group was smaller (n = 149), with many individual serotypes yielding sample sizes too small to ensure model stability. In children old enough to have received one dose, VT IPD (OR = 4.52, adjusted for year of infection, median household size, and residence in the southern federal states) and IPD from the three non-PCV7 serotypes (OR = 13.35) had higher odds of occurring in unvaccinated children, while IPD from serotypes 10A and 15B had higher odds (OR = 7.54 and OR = 11.87, respectively) and of occurring in correctly-vaccinated children. In the post-primary series cohort (n = 74), VT IPD was significantly higher (OR = 7.29, adjusted for per capita income, year of infection, residence in former East Germany, and percentage of children in public daycare) in vaccinated children, while IPD from serotype 15B was significantly associated with correctly-vaccinated children (OR = 10.10). In the post-booster cohort (n = 46), serotype 3 was significantly associated with correctly-vaccinated children, but the confidence interval was very wide (OR 79.47, CI: 2.45–15531.60, adjusted for age of child and percentage of children enrolled in public daycare). For the PCV10 group, variables besides vaccination status which reached statistical significance in at least one of the multivariate models included median household size, year of infection, percentage of children enrolled in public daycare, and residence in North Rhine- Westphalia, though many of these also had wide confidence intervals.

In the PCV13 group (n = 374), in children old enough to have received at least one dose, odds of VT IPD were higher in unvaccinated children across all VT groupings: VT IPD had an OR of 6.21 (adjusted for year of infection, age of child, residence in the southern federal states, per capita income, and percentage of children enrolled in public daycare); PCV10 serotypes had an OR of 7.85; PCV7 serotypes had an OR of 7.65; PCV13non7 types had an OR of 3.03; PCV13non10 types had an OR of 2.13. Among single serotypes, unvaccinated children had higher odds of IPD caused by serotypes 19F, 6A, 6B, and 7F (OR = 3.67, OR = 8.83, OR = 16.05, and OR = 7.48, respectively), and lower odds of IPD in serotype 24F (OR = 0.36). In the same age cohort, correctly vaccinated children were associated with infection from serotype 10A (OR = 3.23). In the post-primary series cohort (n = 177), unvaccinated children had significantly higher odds of VT IPD, PCV7 type IPD, PCV10 type IPD, PCV13non7 IPD, PCV13non10 IPD, as well as IPD from single serotypes 14, and 19A. Unvaccinated children continued to have significantly lower odds of IPD from serotype 24F. Correctly-vaccinated children in this cohort had higher odds of getting IPD from serotype 10A. In the post-booster cohort (n = 147), correctly vaccinated children had higher odds of infection from serotype 15C. Aside from vaccination status, the following variables reached statistical significance in the multivariate models at least once: year of infection, age of child, residence in the northeastern federal states, percentage of unemployed adults, percentage of children enrolled in public daycare, residence in North Rhine- Westphalia, per capita income, median household size, residence in the central federal states, and residence in the southern federal states.

Residence in the former East German federal states or the former West German federal states was not a significant predictor of vaccination status, nor was it a significant predictor for infection with any serotype(s) in the PCV13 group or the PCV10 group. In the PCV7 group, in children old enough to receive one dose, residence in the former East Germany was significantly associated with serotype 19A IPD and serotype 23B IPD, though this association disappeared in the older age cohorts. Demographic variables geographic group and per former political group can be found in **S1 Fig**.

The proportion of non-vaccine serotypes significantly increased over the study period across three of the four geographic groups and in Germany as a whole, however, no single serotype increased significantly when comparing the 2007–2008 season to the 2014–15 season. Some regional differences can still be seen among non-vaccine serotypes. Percentages of selected non-vaccine serotypes by geographic analysis group are shown in **S2 Fig**.

## Discussion

This study shows only the data from children who had IPD, and so making demographic characterizations about the population of German children as a whole is difficult, as we do not know the vaccination status and adherence to the recommended vaccination schedule of children who did not contract IPD. The voluntary nature of IPD reporting in Germany is a continual hurdle for studies using the GNRCS collection. However, the data here show a fairly consistent rate of representation across the federal states, taking population into account.

The variable most often significantly associated with IPD from particular serotypes in the 141 multivariate models generated in this study was vaccination status (23.4%), followed by year of infection and demographic variables (each 14.1%), followed by age (9.9%), with geographic variables providing showing a significant association the least often (in 8.5% of the models). These results show that vaccination status is a strong indicator of VT IPD infection: unvaccinated children had higher odds of VT infections as a whole, and in some individual VT serotypes as well (serotype 19F in the PCV7 group and serotypes 14, 19A, 19F, 6A, 6B, 7F in the PCV13 group), while correctly-vaccinated children had higher odds of non-VT IPD (serotype 12F in the PCV7 group, serotypes 10A, 15B and 3 in the PCV10 group, and serotypes 10A and 15C in the PCV13 group). These results are consistent with several PCV effectiveness studies in countries with PCV programs [10–12] and with serotype distribution studies that have been conducted in countries yet to implement these programs [13–15], which collectively emphasize the importance of vaccination in preventing VT IPD.

Interestingly, no significant differences were found in IPD incidence estimates, serotype distribution or vaccination status between former east and west federal states in this study, which is a departure from previous results [16]. Extending this analysis to the full population of Germany may help establish if this trend is only occurring in young children, or if it can be applied across all age groups.

Some differences between geographic groups are evident, particularly in non-vaccine serotypes, which may indicate that population-normalized regional analysis is useful for determining newly ascendant serotypes and identifying pockets of persistent vaccine serotypes. Upcoming serotypes identified here from the 2014–2015 season include 12F (echoed by studies in France [17, 18], Brazil [19], and Israel [20]) in the central and northeastern states, serotype 38 (also seen in the UK [21], Finland [22], and Hungary [23]) in the northeastern states, serotype 15C (corroborated by results from Canada [24], Uruguay [25], and Korea [26] ) in North Rhine-Westphalia, as well as both the northeastern and southern states, and serotype 10A (increasing in non-invasive isolates in Japan [27] and in IPD in South America [19, 25, 28] in North Rhine-Westphalia, along with the central and northeastern states, all four of which appear in a global review [10] of upcoming serotypes.

Serotypes 7F, 38, 19A, 3, and 33F have been identified [29] as having a high invasive capacity. In the PCV13 group, , these serotypes comprised 26.3% of IPD cases (26.9% in unvaccinated children; 21.7% in correctly-vaccinated children) in children under six during the most recent four seasons, which supports the importance of the role of these serotypes in childhood IPD.

While few IPD studies [30, 31] have included the use of geographic analysis groups, the use of postal codes to approximate socioeconomic status (SES) was recently validated [32] and presents further potential for expanding upon spatial epidemiology studies of IPD in Germany, particularly for adults (preliminary studies using postal codes in children under six had sample sizes too low for model stability), which may soon be of greater interest due to the possible implementation of a PCV recommendation for older adults.

Unvaccinated and incorrectly vaccinated children represent nearly all (94%, 207/220) VT cases of known vaccination status in the post PCV era. While several studies have shown both the 3+1 and 2+1 administration schedules to be highly effective at preventing VT IPD [33, 34] in children, previous work by our group [35] has described troubling laxity in vaccine administration in Germany. While receipt of any PCV dose has been shown to be better than no dose at all [36], the increased effect of being unvaccinated shown here for VT IPD in the post-primary series age cohorts of the PCV10 and PCV13 groups (the PCV7 group lost statistical significance in the post-primary series age cohort) provides additional evidence [37] that more PCV doses provide better protection from VT serotypes.

It is encouraging to see that rates of vaccination are fairly consistent throughout Germany, though the low rates of correctly-vaccinated children are problematic, particularly in light of the recent change to the 2+1 schedule and the anticipated arrival of new vaccines [38], so the situation will need to be carefully monitored.

## Conclusions

PCVs have had a massive impact on IPD cases in German children, playing a major role in both risk of infection and in serotype distribution. Despite a largely uniform population in terms of overall size, rate of vaccination, and several demographic characteristics, there are regional differences in serotype distribution, especially in non-vaccine serotypes. However, the once-prominent distinctions between the former East and West German Republics have blurred, at least in this age group. The importance of continued surveillance remains evident, particularly in light of the recent change to the vaccine administration schedule in Germany and the anticipated arrival of the third-generation pneumococcal conjugate vaccine.

## Supporting information

S1 Fig Distribution of demographic variables from the 2011 census. Variables are displayed by geographic group (left) or by former political group (right).
(TIF)

S2 Fig Rising non-vaccine serotypes following PCV program implementation in Germany. **Cases of** selected non-vaccine serotypes causing IPD in children under six per pneumococcal season, seen across the geographic analysis groups. While the proportion of all non-vaccine serotype IPD increased significantly in three of the four geographic groups and across all of Germany (P = 0.0388 in North Rhine-Westphalia, P = 0.002 in the central states, P = 0.002 in the northeastern states, P = 0.0006 in the southern states, P = 1.30 x 10^−9^ in Germany overall), no individual serotype reached significance when comparing the proportions of non-vaccine serotypes in the first and last years of the study period.
(TIF)

S1 Table Variables influencing pneumococcal vaccination status. Multivariate logistic regression results showing any models with at least one factor which reached statistical significance with vaccination status as the outcome variable and the geographic and demographic variables as potential predictor variables. No variables were significantly associated in the PCV10 group.
(PDF)

S2 Table Logistic regression results for the PCV7 group. Univariate and multivariate ORs and 95% CIs are shown for three age cohorts: at least one dose (≥90 days old), n = 244, post primary series (149–449 days old), n = 110 , and post booster dose (>449 days old), n = 88. Variables that reached statistical significance in the multivariate models appear in bold.
(PDF)

S3 Table Logistic regression results for the PCV10 group. Univariate and multivariate ORs and 95% CIs are shown for three age cohorts: at least one dose (≥90 days old), n = 149, post primary series (149–449 days old), n = 74, and post booster dose (>449 days old), n = 46. Variables that reached statistical significance in the multivariate models appear in bold.
(PDF)

S4 Table Logistic regression results for the PCV13 group. Univariate and multivariate ORs and 95% CIs are shown for three age cohorts: at least one dose (≥90 days old),n = 374, post primary series (149–449 days old), n = 177, and post booster dose (>449 days old), n = 147. Variables that reached statistical significance in the multivariate models appear in bold.
(PDF)

S1 Dataset Full spreadsheet of data used for multiple regression analyses. (XLSX)
