# Supplementary figures and images for: Regional variations in serotype distribution and vaccination status in children under six years of age with invasive pneumococcal disease in Germany
Source: PLoS One. 2019 Jan 9;14(1):e0210278. doi: 10.1371/journal.pone.0210278 (PMC6326516; doi:10.1371/journal.pone.0210278)

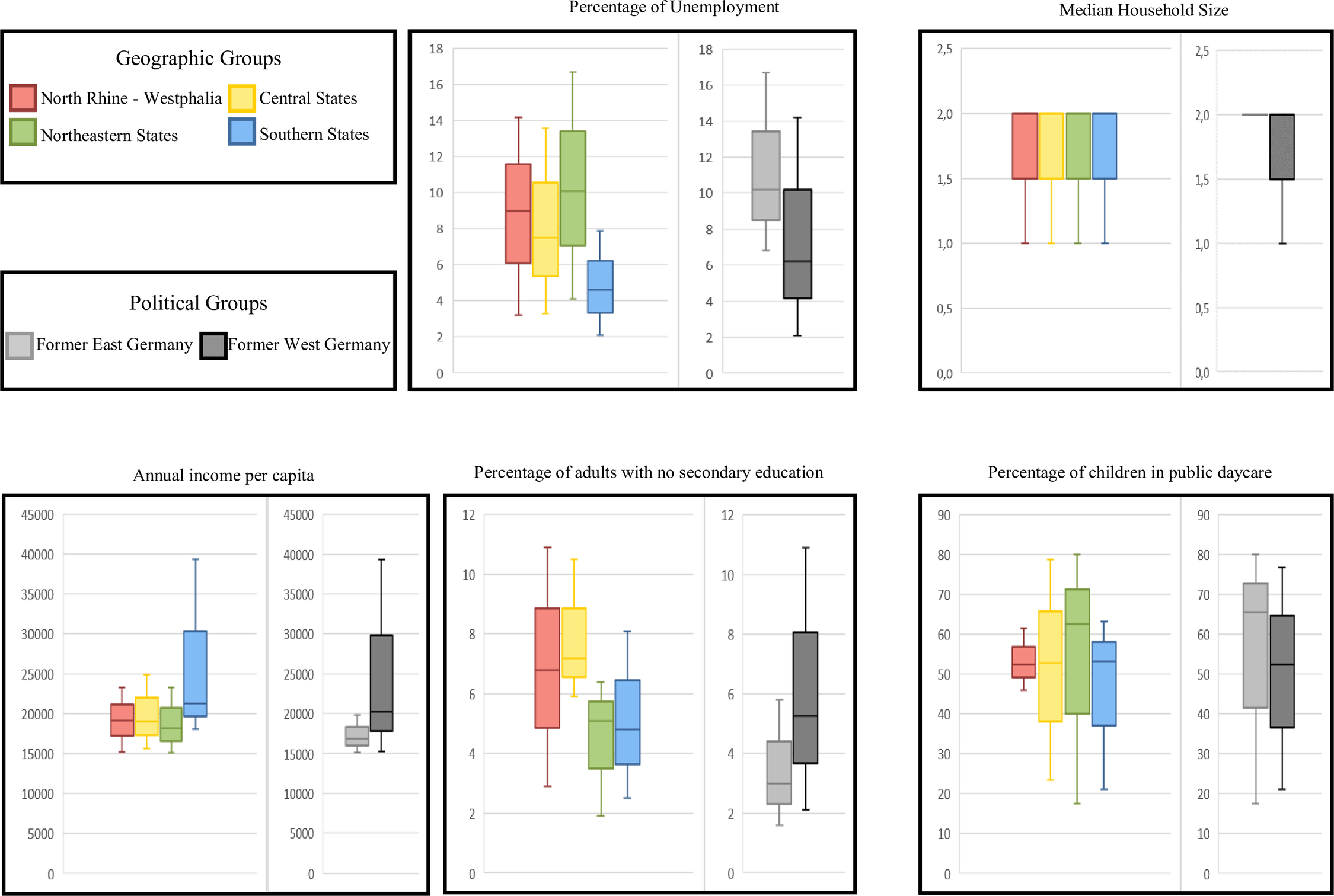

Supplement: S1 Fig — Variables are displayed by geographic group (left) or by former political group (right). (TIF) [file pone.0210278.s001.tif]

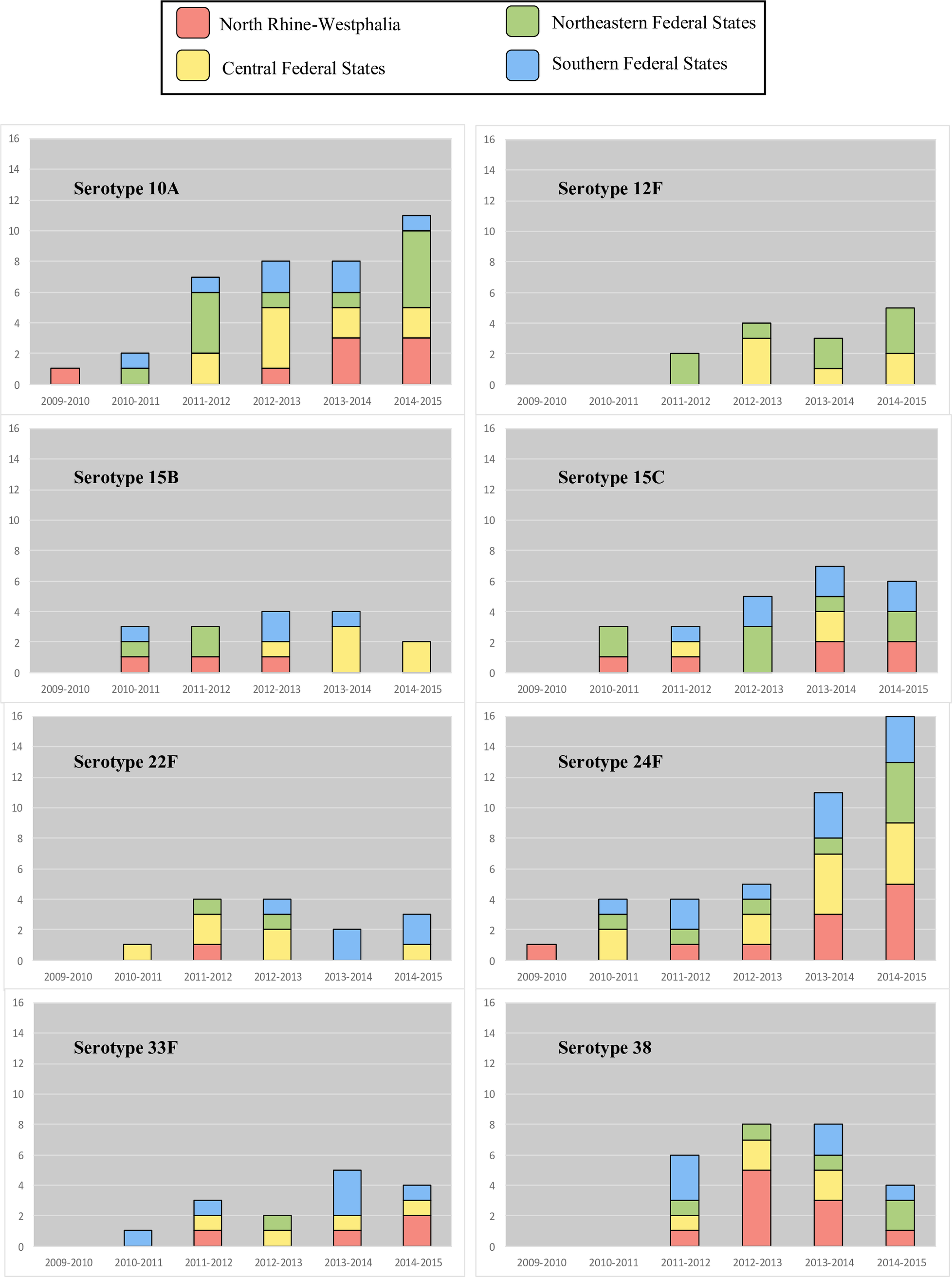

Supplement: S2 Fig — Cases of selected non-vaccine serotypes causing IPD in children under six per pneumococcal season, seen across the geographic analysis groups. While the proportion of all non-vaccine serotype IPD increased significantly in three of the four geographic groups and across all of Germany (P = 0.0388 in North Rhine-Westphalia, P = 0.002 in the central states, P = 0.002 in the northeastern states, P = 0.0006 in the southern states, P = 1.30 x 10^−9^ in Germany overall), no individual serotype reached significance when comparing the proportions of non-vaccine serotypes in the first and last years of the study period. (TIF) [file pone.0210278.s002.tif]
