# Supplementary material for: Regional variations in serotype distribution and vaccination status in children under six years of age with invasive pneumococcal disease in Germany
Source: PLoS One. 2019 Jan 9;14(1):e0210278. doi: 10.1371/journal.pone.0210278 (PMC6326516; doi:10.1371/journal.pone.0210278)
Supplement: S1 Table — Multivariate logistic regression results showing any models with at least one factor which reached statistical significance with vaccination status as the outcome variable and the geographic and demographic variables as potential predictor variables. No variables were significantly associated in the PCV10 group. (PDF) [file pone.0210278.s003.pdf]

at least one dose of PCV7, n = 244							
Univariate model				Multivariate model			
	OR	95% CI			OR	95% CI	
Unvaccinated				Unvaccinated		n = 137	
Year of Infection	1.56	1.34	1.85	**Year of Infection**	**9.68**	**2.85**	**20791.29**
Age of Child	0.55	0.43	0.70	Age of Child	0.08	3.13×10^{-5}	148.83
Correctly Vaccinated				Correctly Vaccinated		n = 23	
Year of Infection	0.67	0.49	0.88	Year of Infection	0.72	0.03	1.16
No Secondary Education	4.84	1.31	95.38	**No Secondary Education**	**4.22**	**1.15**	**67.35**
Former East Germany	1.95	0.69	4.95	Former East Germany	6.67	0.01	2951.98
at least one dose of PCV13, n = 374							
Univariate model				Multivariate model			
	OR	95% CI			OR	95% CI	
Unvaccinated				Unvaccinated		n = 102	
North Rhine Westphalia	0.65	0.34	1.17	North Rhine Westphalia	0.73	0.37	1.36
Income per capita	1.00	1.00	1.00	**Income per capita**	**1.00**	**1.00**	**1.00**
Northeastern States	0.88	0.51	1.48	Northeastern States	0.90	0.47	1.69
PCV13 post-primary series, n = 177							
Univariate model				Multivariate model			
	OR	95% CI			OR	95% CI	
Correctly Vaccinated				Correctly Vaccinated		n = 38	
North Rhine Westphalia	0.76	0.29	1.76	North Rhine Westphalia	0.77	0.28	1.95
Southern States	0.63	0.24	1.45	Southern States	0.99	0.32	3.02
Income per capita	0.99	0.99	0.99	Income per capita	0.99	0.99	1.00
Daycare Use	1.01	1.00	1.06	**Daycare Use**	**1.00**	**1.00**	**1.04**
PCV13 post-booster, n = 147							
Univariate model				Multivariate model			
	OR	95% CI			OR	95% CI	
Unvaccinated				Unvaccinated		n = 29	
Central States	0.92	0.33	2.32	Central States	1.81	0.56	5.75
Southern States	2.99	1.32	6.90	**Southern States**	**3.67**	**1.44**	**10.08**
