# Supplementary material for: Regional variations in serotype distribution and vaccination status in children under six years of age with invasive pneumococcal disease in Germany
Source: PLoS One. 2019 Jan 9;14(1):e0210278. doi: 10.1371/journal.pone.0210278 (PMC6326516; doi:10.1371/journal.pone.0210278)
Supplement: S2 Table — Univariate and multivariate ORs and 95% CIs are shown for three age cohorts: at least one dose (≥90 days old), n = 244, post primary series (149–449 days old), n = 110 , and post booster dose (>449 days old), n = 88. Variables that reached statistical significance in the multivariate models appear in bold. (PDF) [file pone.0210278.s004.pdf]

at least one dose of PCV7, n= 244							
Univariate model				Multivariate model			
Serotype(s)	OR	95% CI		Serotype(s)	OR	95% CI	
PCV7types				PCV7types		n =36	
Unvaccinated	7.09	2.80	22.67	**Unvaccinated**	**6.84**	**2.66**	**22.06**
Income per capita	1.00	0.99	1.00	Income per capita	1.00	0.99	1.00
Northeastern States	0.28	0.07	0.78	**Northeastern States**	**0.29**	**0.06**	**0.97**
15B				15B		n = 6	
Unvaccinated	1.42	0.31	8.28	Unvaccinated	1.52	0.33	8.99
North Rhine Westphalia	5.05	1.04	24.64	**North Rhine Westphalia**	**5.12**	**1.05**	**24.99**
15C				15C		n = 14	
Age of Child	1.37	0.93	1.93	Age of Child	1.38	0.93	2.00
Unemployment	1.13	0.94	1.36	Unemployment	1.00	0.98	1.03
Southern States	0.19	0.02	0.81	**Southern States**	**0.20**	**0.02**	**0.93**
North Rhine Westphalia	2.99	0.92	8.84	North Rhine Westphalia	1.53	0.43	5.02
19A				19A		n = 39	
Northeastern States	0.16	0.10	0.24	Northeastern States	1.57	0.57	4.02
Former East Germany	3.17	1.45	6.77	**Former East Germany**	**4.18**	**1.46**	**11.74**
Household Size	0.26	0.10	0.74	**Household Size**	**0.18**	**0.07**	**0.54**
19F				19F		n = 13	
Unvaccinated	7.07	1.67	65.66	**Unvaccinated**	**7.17**	**1.68**	**66.73**
Northeastern States	0.36	0.04	1.52	Northeastern States	0.43	0.04	2.06
Central States	2.16	0.66	6.49	Central States	1.81	0.53	5.84
23B				23B		n = 4	
Unvaccinated	7.25	0.76	964.35	Unvaccinated	3.84	0.29	548.12
Former East Germany	5.10	0.77	33.93	**Former East Germany**	**8.84**	**1.14**	**86.65**
Year of Infection	1.70	1.04	3.08	Year of Infection	1.69	0.96	3.75
38				38		n = 6	
No Secondary Education	0.46	0.10	1.17	No Secondary Education	0.79	0.45	1.30
Daycare Use	1.00	0.95	1.01	**Daycare Use**	**0.93**	**0.88**	**0.99**
6B				6B		n = 8	
Unvaccinated	4.08	0.87	39.12	Unvaccinated	2.88	0.52	31.83
Unemployment	0.59	0.32	0.91	Unemployment	0.95	0.86	1.02
Income per capita	1.00	1.00	1.00	Income per capita	1.00	0.99	1.00
Age of Child	0.54	0.13	1.16	Age of Child	0.68	0.17	1.46
Southern States	10.10	2.15	96.91	**Southern States**	**15.62**	**1.47**	**2323.12**

PCV7 post-primary series, n = 110							
Univariate model				Multivariate model			
Serotype(s)	OR	95% CI		Serotype(s)	OR	95% CI	
10A				10A		n = 6	
Income per capita	1.00	1.00	1.00	**Income per capita**	**1.00**	**1.00**	**1.00**
No Secondary Education	0.38	0.13	0.82	No Secondary Education	0.31	0.07	0.82
12F				12F		n = 3	
Correctly Vaccinated	11.18	0.90	100.97	**Correctly Vaccinated**	**15.52**	**1.03**	**293.10**
Year of Infection	1.21	0.72	1.98	Year of Infection	1.37	0.78	2.57

Age of Child	1.60	0.14	12.51	Age of Child	1.13	0.09	9.12
15C				15C		n = 4	
Central States	3.76	0.55	25.60	Central States	2.25	0.30	16.27
Income per capita	0.99	0.99	0.99	**Income per capita**	**0.99**	**0.99**	**0.99**
19A				19A		n = 22	
Age of Child	2.21	0.83	5.77	Age of Child	1.51	0.50	4.33
Former East Germany	2.29	0.79	6.35	Former East Germany	2.40	0.68	7.89
Household Size	0.21	0.04	1.09	**Household Size**	**0.17**	**0.03**	**0.91**
19F				19F		n = 6	
Unvaccinated	8.16	0.92	1075.18	**Unvaccinated**	**10.49**	**1.07**	**1443.67**
Central States	3.88	0.77	19.58	Central States	2.94	0.49	16.56
Daycare Use	1.12	1.02	1.25	**Daycare Use**	**1.12**	**1.01**	**1.26**
24F				24F		n = 2	
Age of Child	14.12	1.11	1971.00	**Age of Child**	**15.35**	**1.18**	**2178.40**
North Rhine Westphalia	5.61	0.44	72.30	North Rhine Westphalia	6.99	0.49	105.66
PCV7 post-booster, n = 88							
Univariate model				Multivariate model			
Serotype(s)	OR	95% CI		Serotype(s)	OR	95% CI	
PCV7types				PCV7types		n = 7	
Unvaccinated	8.59	1.69	85.42	**Unvaccinated**	**5.72**	**1.05**	**57.62**
Southern States	4.36	0.98	25.54	Southern States	2.83	0.58	17.97
Daycare Use	0.95	0.90	1.01	Daycare Use	0.95	0.89	1.02
1				1		n = 8	
Unvaccinated	0.30	0.03	1.46	Unvaccinated	0.26	0.02	1.55
Central States	5.70	1.38	26.80	**Central States**	**5.73**	**1.09**	**43.56**
Age of Child	1.66	0.93	3.03	**Age of Child**	**1.94**	**1.04**	**3.94**
North Rhine Westphalia	0.21	0.00	1.87	North Rhine Westphalia	0.24	0.00	2.80
Daycare Use	0.96	0.91	1.02	Daycare Use	0.97	0.91	1.03
19A				19A		n = 14	
Correctly Vaccinated	3.21	0.68	13.22	Correctly Vaccinated	2.42	0.36	13.48
Household Size	0.29	0.07	1.40	**Household Size**	**0.17**	**0.03**	**0.89**
Former East Germany	3.20	0.90	10.80	Former East Germany	3.94	0.57	25.90
Northeastern States	2.89	0.82	9.65	Northeastern States	4.93	0.77	30.46
Year of Infection	0.74	0.47	1.11	Year of Infection	0.88	0.48	1.41
Age of Child	0.64	0.34	1.09	Age of Child	0.66	0.29	1.45
24F				24F		n = 5	
No Secondary Education	1.52	0.95	2.55	No Secondary Education	1.45	0.89	2.51
Year of Infection	2.34	1.29	4.83	**Year of Infection**	**2.18**	**1.19**	**4.55**
38				38		n = 4	
Central States	7.84	1.21	84.48	Central States	4.90	0.66	54.79
Daycare Use	0.92	0.86	0.98	**Daycare Use**	**0.93**	**0.87**	**0.99**
Age of Child	0.41	0.06	1.18	Age of Child	0.42	0.07	1.26
