# Supplementary material for: Regional variations in serotype distribution and vaccination status in children under six years of age with invasive pneumococcal disease in Germany
Source: PLoS One. 2019 Jan 9;14(1):e0210278. doi: 10.1371/journal.pone.0210278 (PMC6326516; doi:10.1371/journal.pone.0210278)
Supplement: S3 Table — Univariate and multivariate ORs and 95% CIs are shown for three age cohorts: at least one dose (≥90 days old), n = 149, post primary series (149–449 days old), n = 74, and post booster dose (>449 days old), n = 46. Variables that reached statistical significance in the multivariate models appear in bold. (PDF) [file pone.0210278.s005.pdf]

at least one dose of PCV10, n = 149							
Univariate model				Multivariate model			
Serotype(s)	OR	95% CI		Serotype(s)	OR	95% CI	
PCV7types				PCV7types		n = 28	
Northeastern States	0.38	0.10	1.11	Northeastern States	0.24	0.03	1.03
Household Size	6.13	0.75	795.55	**Household Size**	**9.29**	**1.11**	**1215.05**
Income per capita	1.00	0.99	1.00	Income per capita	1.00	0.99	1.00
PCV10types				PCV10types		n = 43	
Unvaccinated	3.05	1.22	9.02	**Unvaccinated**	**4.52**	**1.60**	**15.62**
Year of Infection	0.70	0.54	0.89	**Year of Infection**	**0.67**	**0.51**	**0.86**
Household Size	3.32	0.74	31.46	**Household Size**	**6.01**	**1.20**	**60.97**
Southern States	1.60	0.78	3.29	Southern States	1.38	0.62	3.07
PCV10non7				PCV10non7		n = 15	
Unvaccinated	11.92	1.53	1535.99	**Unvaccinated**	**13.35**	**1.70**	**1725.59**
Year of Infection	0.78	0.53	1.09	Year of Infection	0.74	0.50	1.05
10A				10A		n = 8	
Correctly Vaccinated	9.55	1.50	49.89	**Correctly Vaccinated**	**7.54**	**1.02**	**47.46**
Age of Child	0.36	0.04	1.13	Age of Child	0.36	0.04	1.08
Northeastern States	3.51	0.59	14.34	Northeastern States	1.73	0.30	9.88
Southern States	0.33	0.03	1.58	Southern States	0.44	0.04	2.95
Unemployment	0.85	0.64	1.05	Unemployment	0.99	0.94	1.03
12F				12F		n = 6	
Unvaccinated	0.32	0.06	1.55	Unvaccinated	0.25	0.05	1.31
Year of Infection	1.73	1.06	3.13	**Year of Infection**	**1.82**	**1.11**	**3.34**
Central States	0.20	0.00	1.73	Central States	0.15	0.00	1.75
Northeastern States	3.43	0.70	16.92	Northeastern States	2.70	0.47	15.62
Unemployment	1.17	0.93	1.46	Unemployment	1.02	0.97	1.07
15B				15B		n = 6	
Correctly Vaccinated	13.99	2.08	81.36	**Correctly Vaccinated**	**11.87**	**1.73**	**68.73**
Central States	2.84	0.58	13.95	Central States	2.39	0.46	12.15
19F				19F		n = 13	
Year of Infection	0.88	0.43	0.48	**Year of Infection**	**0.67**	**0.41**	**0.99**
Daycare Use	1.08	1.00	1.16	**Daycare Use**	**1.07**	**1.00**	**1.15**
7F				7F		n = 10	
Unvaccinated	7.68	0.95	997.00	Unvaccinated	8.77	0.97	1175.37
Year of Infection	0.57	0.30	0.91	**Year of Infection**	**0.55**	**0.29**	**0.91**
Central States	0.12	0.00	0.95	Central States	0.18	0.00	1.52
Unemployment	1.10	0.97	1.24	Unemployment	0.99	0.95	1.03

PCV10 post-primary series, n =74							
Univariate model				Multivariate model			
Serotype(s)	OR	95% CI		Serotype(s)	OR	95% CI	
PCV10types				PCV10types		n = 19	
Unvaccinated				**Unvaccinated**	**7.29**	**1.40**	**85.26**
Income per capita	0.99	0.99	1.00	Income per capita	0.99	0.99	1.00
Year of Infection	0.76	0.53	1.04	Year of Infection	0.74	0.49	1.06
Former East Germany	2.69	0.80	8.87	Former East Germany	0.99	0.09	10.05
Daycare Use	1.07	1.00	1.15	Daycare Use	1.06	0.95	1.18

14				14	n = 2		
Year of Infection	8.47	1.35	1308.65	**Year of Infection**	**3.50**	**1.11**	**568.63**
North Rhine Westphalia	0.73	0.65	118.72	North Rhine Westphalia	5.16	0.16	103.72
15B				15B	n = 5		
Correctly Vaccinated	10.40	1.43	71.24	**Correctly Vaccinated**	**10.10**	**1.23**	**87.84**
Central States	3.63	0.65	23.26	Central States	1.38	0.20	9.59
Northeastern States	0.18	0.00	1.70	Northeastern States	0.17	0.00	2.44
19F				19F	n = 7		
Year of Infection	0.49	0.20	0.89	**Year of Infection**	**0.46**	**0.12**	**0.87**
Daycare Use	1.12	1.02	1.25	**Daycare Use**	**1.12**	**1.02**	**1.24**
6A				6A	n = 2		
Household Size	0.02	0.00	0.41	**Household Size**	**0.05**	**0.00**	**0.90**
Income per capita	1.00	0.99	1.00	Income per capita	1.00	0.99	1.00
PCV10 post-booster, n = 46							
Univariate model				Multivariate model			
Serotype(s)	OR	95% CI		Serotype(s)	OR	95% CI	
19A				19A	n = 6		
Year of Infection	0.29	0.08	0.75	Year of Infection	0.42	0.01	1.54
Age of Child	0.23	0.00	0.89	Age of Child	0.30	0.01	4.26
North Rhine Westphalia	15.40	2.27	128.30	**North Rhine Westphalia**	**59.96**	**2.31**	**5.03 x 10^9**
Household Size	0.08	0.01	0.53	**Household Size**	**0.02**	**0.00**	**0.45**
24F				24F	n = 3		
Year of Infection	5.32	4.63	540.27	**Year of Infection**	**4.59**	**1.57**	**634.34**
North Rhine Westphalia	5.27	0.42	50.26	North Rhine Westphalia	3.66	0.17	102.76
3				3	n = 4		
Correctly Vaccinated	36.43	1.64	5759.21	**Correctly Vaccinated**	**79.47**	**2.45**	**15531.60**
Age of Child	1.87	0.86	4.39	Age of Child	1.64	0.65	4.33
Daycare Use	0.94	0.87	1.02	Daycare Use	0.92	0.83	1.00
