# Supplementary material for: Regional variations in serotype distribution and vaccination status in children under six years of age with invasive pneumococcal disease in Germany
Source: PLoS One. 2019 Jan 9;14(1):e0210278. doi: 10.1371/journal.pone.0210278 (PMC6326516; doi:10.1371/journal.pone.0210278)
Supplement: S4 Table — Univariate and multivariate ORs and 95% CIs are shown for three age cohorts: at least one dose (≥90 days old),n = 374, post primary series (149–449 days old), n = 177, and post booster dose (>449 days old), n = 147. Variables that reached statistical significance in the multivariate models appear in bold. (PDF) [file pone.0210278.s006.pdf]

at least one dose of PCV13, n = 374							
Univariate model				Multivariate model			
Serotype(s)	OR	95% CI		Serotype(s)	OR	95% CI	
PCV7types				PCV7types		n = 26	
Unvaccinated	10.19	4.28	27.55	**Unvaccinated**	**7.65**	**3.01**	**21.66**
Year of Infection	0.65	0.49	0.86	Year of Infection	0.84	0.62	1.12
North Rhine Westphalia	0.40	0.08	1.26	North Rhine Westphalia	0.44	0.08	1.80
Northeastern States	0.41	0.11	1.16	Northeastern States	0.40	0.09	1.46
Southern States	2.91	1.32	6.51	Southern States	1.41	0.53	3.94
PCV10types				PCV10types		n = 43	
Unvaccinated	12.05	5.92	26.46	**Unvaccinated**	**7.85**	**3.68**	**17.82**
Year of Infection	0.53	0.41	0.67	**Year of Infection**	**0.68**	**0.52**	**0.87**
Southern States	2.00	1.04	3.79	Southern States	1.67	0.80	3.42
PCV13types				PCV13types		n = 104	
Unvaccinated	7.31	4.43	12.24	**Unvaccinated**	**6.21**	**3.45**	**11.36**
Year of Infection	0.60	0.51	0.71	**Year of Infection**	**0.64**	**0.52**	**0.79**
Age of Child	1.24	0.99	1.55	**Age of Child**	**1.74**	**1.31**	**2.32**
Southern States	1.90	1.18	3.05	Southern States	1.82	0.95	3.50
Income per capita	1.00	1.00	1.00	Income per capita	0.99	0.99	1.00
Daycare Use	0.98	0.96	1.00	Daycare Use	0.99	0.96	1.01
PCV13non7				PCV13non7		n = 78	
Unvaccinated	3.95	2.35	6.68	**Unvaccinated**	**3.03**	**1.71**	**5.36**
Year of Infection	0.66	0.55	0.78	**Year of Infection**	**0.70**	**0.57**	**0.85**
Age of Child	1.20	0.94	1.52	**Age of Child**	**1.46**	**1.12**	**1.91**
PCV13non10				PCV13non10		n = 61	
Unvaccinated	2.52	1.43	4.42	**Unvaccinated**	**2.13**	**1.10**	**4.09**
Year of Infection	0.78	0.65	0.94	Year of Infection	0.83	0.66	1.04
Age of Child	1.33	1.03	1.70	**Age of Child**	**1.50**	**1.13**	**1.98**
Central States	0.64	0.31	1.23	Central States	0.73	0.32	1.60
Southern States	1.51	0.85	2.65	Southern States	1.24	0.63	2.45
Household Size	0.50	0.20	1.39	Household Size	0.60	0.23	1.75
10A				10A		n = 37	
Correctly Vaccinated	3.58	1.74	7.25	**Correctly Vaccinated**	**3.23**	**1.50**	**6.83**
Year of Infection	1.17	0.93	1.51	Year of Infection	1.21	0.92	1.60
Age of Child	0.66	0.40	0.99	Age of Child	0.68	0.40	1.06
Southern States	0.53	0.22	1.17	Southern States	0.60	0.23	1.38
Unemployment	0.99	0.97	1.01	Unemployment	0.99	0.98	1.00
Household Size	0.46	0.17	1.54	Household Size	0.45	0.15	1.59
12F				12F		n = 18	
Year of Infection	1.32	0.94	1.94	**Year of Infection**	**1.42**	**1.00**	**2.11**
North Rhine Westphalia	0.34	0.04	1.37	North Rhine Westphalia	0.44	0.05	2.04
Northeastern States	2.49	0.95	6.36	**Northeastern States**	**2.84**	**1.03**	**7.89**
Unemployment	1.01	0.99	1.03	**Unemployment**	**1.02**	**1.00**	**1.04**
Daycare Use	1.01	1.00	1.02	**Daycare Use**	**1.01**	**1.01**	**1.02**
15C				15C		n = 24	
Correctly Vaccinated	1.98	0.76	4.70	Correctly Vaccinated	2.26	0.84	5.64
Age of Child	1.54	1.08	2.14	**Age of Child**	**1.61**	**1.12**	**2.28**

Central States	0.47	0.12	1.32	Central States	0.48	0.12	1.36
Unemployment	0.99	0.98	1.01	Unemployment	0.99	0.98	1.01
19F				19F		n = 15	
Unvaccinated	5.52	1.98	17.18	**Unvaccinated**	**3.67**	**1.15**	**12.53**
Year of Infection	0.64	0.44	0.91	Year of Infection	0.69	0.44	1.03
Age of Child	1.43	0.91	2.14	Age of Child	1.59	0.99	2.50
Central States	2.13	0.73	5.88	Central States	3.28	0.64	32.52
Northeastern States	0.29	315.00	1.20	Northeastern States	0.68	0.05	8.59
Southern States	2.10	0.75	5.80	Southern States	2.36	0.48	23.09
24F				24F		n = 41	
Unvaccinated	0.29	0.09	0.71	**Unvaccinated**	**0.36**	**0.11**	**0.93**
Year of Infection	1.37	1.08	1.78	Year of Infection	1.25	0.97	1.65
North Rhine Westphalia	1.64	0.76	3.33	North Rhine Westphalia	1.40	0.64	2.89
Unemployment	1.00	0.99	1.01	Unemployment	1.00	0.99	1.02
3				3		n = 20	
Correctly Vaccinated	0.32	0.04	1.30	Correctly Vaccinated	0.42	0.05	1.72
Age of Child	1.81	1.27	2.57	**Age of Child**	**1.73**	**1.20**	**2.46**
Southern States	1.98	0.80	4.82	Southern States	1.75	0.59	4.34
38				38		n = 26	
Unvaccinated	0.25	0.05	0.79	Unvaccinated	0.27	0.05	0.86
North Rhine Westphalia	2.86	1.23	6.43	**North Rhine Westphalia**	**2.66**	**1.13**	**6.02**
6A				6A		n = 7	
Unvaccinated	12.19	2.52	118.05	**Unvaccinated**	**8.83**	**1.36**	**107.97**
Age of Child	1.93	1.10	3.24	**Age of Child**	**2.48**	**1.30**	**5.10**
Central States	0.20	0.00	1.63	Central States	1.06	0.01	207.16
Southern States	5.26	1.25	29.63	Southern States	9.68	0.93	1313.81
Income per capita	1.00	0.99	1.00	Income per capita	1.00	0.99	1.00
6B				6B		n = 5	
Unvaccinated	30.74	3.44	4049.81	**Unvaccinated**	**16.05**	**1.34**	**2230.15**
Year of Infection	0.55	0.27	0.98	Year of Infection	0.98	0.48	2.01
Age of Child	0.39	0.04	1.37	Age of Child	0.58	0.08	1.70
Southern States	7.14	1.30	71.77	Southern States	9.85	0.69	3086.16
Unemployment	0.99	0.96	1.02	Unemployment	0.95	0.80	1.03
Income per capita	1.00	1.00	1.00	Income per capita	1.00	0.99	1.00
7F				7F		n = 9	
Unvaccinated	16.28	3.60	154.35	**Unvaccinated**	**7.48**	**1.41**	**76.29**
Year of Infection	0.37	0.19	0.62	**Year of Infection**	**0.57**	**0.30**	**0.95**
Age of Child	0.51	0.14	1.21	Age of Child	0.77	0.22	1.76
Central States	0.15	0.00	1.23	Central States	0.18	0.00	1.58
Unemployment	1.01	0.98	1.03	Unemployment	0.99	0.97	1.03
PCV13 post primary series, n = 177							
Univariate model				Multivariate model			
Serotype(s)	OR	95% CI		Serotype(s)	OR	95% CI	
PCV7types				PCV7types		n = 12	
Unvaccinated	13.45	3.70	71.99	**Unvaccinated**	**9.37**	**2.35**	**52.62**
Year of Infection	0.60	0.39	0.89	Year of Infection	0.82	0.53	1.22
Age of Child	0.25	0.03	1.07	Age of Child	0.32	0.03	1.49

Southern States	2.27	0.68	7.19	Southern States	1.78	0.47	6.27
PCV10types				PCV10types		n = 19	
Unvaccinated	12.43	4.37	42.66	**Unvaccinated**	**7.49**	**2.47**	**26.57**
Year of Infection	0.54	0.38	0.76	Year of Infection	0.72	0.49	1.02
Age of Child	0.40	0.10	1.17	Age of Child	0.50	0.12	1.66
North Rhine Westphalia	0.25	0.03	1.02	North Rhine Westphalia	0.38	0.04	1.87
Former East Germany	2.08	0.66	5.84	Former East Germany	1.36	0.36	4.67
PCV13types				PCV13types		n = 43	
Unvaccinated	11.36	5.29	25.59	**Unvaccinated**	**8.10**	**3.66**	**18.52**
Year of Infection	0.58	0.45	0.74	**Year of Infection**	**0.70**	**0.53**	**0.92**
North Rhine Westphalia	0.50	0.19	1.20	North Rhine Westphalia	0.77	0.25	2.20
Former East Germany	1.86	0.78	4.26	Former East Germany	1.43	0.49	4.05
PCV13non7				PCV13non7		n = 31	
Unvaccinated	5.96	2.67	13.77	**Unvaccinated**	**4.46**	**1.91**	**10.64**
Year of Infection	0.65	0.49	0.84	Year of Infection	0.77	0.57	1.02
Northeastern States	1.76	0.76	3.94	Northeastern States	1.42	0.55	3.38
PCV13non10				PCV13non10		n = 24	
Unvaccinated	4.61	1.93	11.37	**Unvaccinated**	**4.24**	**1.66**	**11.18**
Year of Infection	0.73	0.54	0.97	Year of Infection	0.82	0.60	1.11
Age of Child	1.79	0.77	4.07	Age of Child	2.35	0.95	5.79
10A				10A		n = 27	
Correctly Vaccinated	5.55	2.35	13.26	**Correctly Vaccinated**	**6.06**	**2.44**	**15.47**
Year of Infection	1.33	0.99	1.82	**Year of Infection**	**1.40**	**1.00**	**2.01**
Household Size	0.35	0.10	1.54	Household Size	0.25	0.06	1.21
12F				12F		n = 9	
Age of Child	0.35	0.04	1.57	Age of Child	0.38	0.04	1.81
North Rhine Westphalia	0.17	0.00	1.37	North Rhine Westphalia	0.25	0.00	2.44
Northeastern States	2.44	0.63	8.99	Northeastern States	2.02	0.50	8.20
Daycare Use	1.01	1.00	1.02	**Daycare Use**	**1.00**	**1.00**	**1.02**
14				14		n = 2	
Unvaccinated	13.53	1.08	1879.00	**Unvaccinated**	**41.89**	**2.70**	**6387.79**
Year of Infection	3.70	0.93	358.46	**Year of Infection**	**8.37**	**1.27**	**1403.91**
19A				19A		n = 19	
Unvaccinated	3.34	1.29	8.79	**Unvaccinated**	**3.28**	**1.17**	**9.37**
Year of Infection	0.78	0.57	1.07	Year of Infection	0.85	0.31	1.19
Age of Child	2.31	0.93	5.69	**Age of Child**	**2.85**	**1.10**	**7.49**
19F				19F		n = 8	
Unvaccinated	7.56	1.85	42.37	Unvaccinated	3.47	0.69	21.43
Year of Infection	0.43	0.23	0.73	**Year of Infection**	**0.57**	**0.30**	**0.95**
North Rhine Westphalia	0.19	0.00	1.57	North Rhine Westphalia	0.33	0.00	3.51
Southern States	3.10	0.77	12.51	Southern States	1.99	0.44	8.76
24F				24F		n = 19	
Unvaccinated	0.18	0.02	0.76	**Unvaccinated**	**0.16**	**0.02**	**0.70**
Income per capita	1.00	0.99	1.00	**Income per capita**	**1.00**	**1.00**	**1.00**
6A				6A		n = 2	
Unvaccinated	13.53	1.08	1879.00	Unvaccinated	4.48	0.08	725.39
Year of Infection	0.41	0.09	1.03	Year of Infection	0.56	0.03	1.63

Former East Germany	5.18	0.41	65.46	Former East Germany	3.80	0.02	704.04
Household Size	0.02	0.00	0.40	**Household Size**	**0.03**	**0.00**	**0.79**
PCV13 post-booster, n = 147							
Univariate model				Multivariate model			
Serotype(s)	OR	95% CI		Serotype(s)	OR	95% CI	
PCV7types				PCV7types		n = 9	
Unvaccinated	5.71	1.51	22.73	Unvaccinated	3.13	0.74	13.82
Age of Child	2.60	1.46	4.97	**Age of Child**	**2.55**	**1.38**	**5.18**
Southern States	3.94	1.07	17.22	Southern States	2.96	0.66	16.42
Former East Germany	0.15	0.00	1.26	Former East Germany	0.95	0.01	20.73
Daycare Use	0.94	0.90	0.99	Daycare Use	0.95	0.89	1.02
PCV10types				PCV10types		n = 12	
Unvaccinated	6.88	2.11	23.84	Unvaccinated	2.79	0.49	15.09
Year of Infection	0.65	0.40	1.02	Year of Infection	0.54	0.23	1.06
Age of Child	2.69	1.59	4.84	**Age of Child**	**35.34**	**1.81**	**8.32**
North Rhine Westphalia	0.19	0.00	1.55	North Rhine Westphalia	0.09	0.00	1.27
Southern States	2.90	0.92	9.74	Southern States	0.70	0.15	3.09
Former East Germany	0.11	0.00	0.90	Former East Germany	0.11	0.00	1.60
Daycare Use	0.95	0.91	0.99	Daycare Use	1.00	0.94	1.08
PCV13types				PCV13types		n = 41	
Unvaccinated	4.48	1.94	10.57	Unvaccinated	1.97	0.62	6.09
Year of Infection	0.68	0.51	0.90	**Year of Infection**	**0.53**	**0.33**	**0.81**
Age of Child	1.90	1.32	2.79	**Age of Child**	**2.96**	**1.80**	**5.28**
Central States	0.42	0.14	1.05	Central States	0.25	0.05	1.07
Southern States	3.18	1.52	6.75	Southern States	1.31	0.43	4.02
Former East Germany	0.38	0.13	0.95	Former East Germany	0.81	0.06	6.96
Income per capita	1.00	1.00	1.00	Income per capita	1.00	0.99	1.00
Daycare Use	0.96	0.92	0.99	Daycare Use	0.96	0.90	1.01
PCV13non7				PCV13non7		n = 32	
Unvaccinated	2.82	1.16	6.72	Unvaccinated	1.39	0.43	4.20
Year of Infection	0.68	0.50	0.92	**Year of Infection**	**0.62**	**0.40**	**0.93**
Age of Child	1.41	0.96	2.06	**Age of Child**	**1.85**	**1.16**	**3.02**
Central States	0.33	0.09	0.97	Central States	0.23	0.04	0.99
Southern States	2.36	1.07	5.24	Southern States	1.30	0.45	3.77
Income per capita	1.00	0.99	1.00	Income per capita	0.99	0.99	1.00
Daycare Use	0.97	0.94	1.01	Daycare Use			
PCV13non10				PCV13non10		n = 29	
Unvaccinated	2.23	0.88	5.44	Unvaccinated	1.26	0.40	3.70
Year of Infection	0.76	0.55	1.03	Year of Infection	0.85	0.58	1.23
Central States	0.25	0.05	0.83	**Central States**	**0.19**	**0.02**	**0.92**
Southern States	2.51	1.11	5.75	Southern States	1.37	0.49	4.01
Former East Germany	0.65	0.22	1.69	Former East Germany	0.83	0.07	6.11
Household Size	0.42	0.13	1.57	Household Size	0.39	0.07	2.06
Daycare Use	0.97	0.94	1.01	Daycare Use	0.97	0.91	1.03
15C				15C		n = 16	
Correctly Vaccinated	5.53	1.59	18.10	**Correctly Vaccinated**	**4.75**	**1.34**	**15.77**
Year of Infection	1.38	0.91	2.19	Year of Infection	1.27	0.83	2.03

19A				19A		n = 9	
Year of Infection	0.62	0.35	1.02	Year of Infection	0.78	0.42	1.40
Household Size	0.11	0.02	0.54	**Household Size**	**0.14**	**0.03**	**0.70**
Income per capita	1.00	0.99	1.00	Income per capita	1.00	0.99	1.00
19F				19F		n = 5	
Age of Child	3.98	1.82	11.58	**Age of Child**	**3.91**	**1.78**	**11.24**
Daycare Use	0.95	0.90	1.03	Daycare Use	0.96	0.89	1.03
24F				24F		n = 21	
Year of Infection	1.65	1.12	2.60	**Year of Infection**	**1.58**	**1.06**	**2.49**
North Rhine Westphalia	2.62	0.87	7.29	North Rhine Westphalia	2.06	0.66	5.90
6A				6A		n = 5	
Unvaccinated	13.82	2.43	142.25	Unvaccinated	7.70	0.98	118.47
Age of Child	1.89	0.89	3.93	Age of Child	2.53	0.11	7.31
Southern States	6.23	1.11	63.39	**Southern States**	**14.86**	**1.26**	**2131.21**
Income per capita	1.00	0.99	1.00	Income per capita	1.00	0.99	1.00
